# *Ximenia americana*: Economic Importance, Medicinal Value, and Current Status in Ethiopia

**DOI:** 10.1155/2021/8880021

**Published:** 2021-03-08

**Authors:** Getachew Amare Kefelegn, Bizuayehu Desta

**Affiliations:** Department of Horticulture, College of Agriculture and Natural Resources Sciences, Debre Berhan University, P.O. Box: 445, Debre Berhan, Ethiopia

## Abstract

*Ximenia americana* is one of the most valuable wild edible plants in the world. In different countries, it is utilized as food, medicine, an essential oil source, and the industrial component to other products. In Ethiopia, it was one of the most known and very important plants for a long period of time. It was utilized as food, a medicinal plant, and animal feed. It was also one of the most economically important and culturally valuable plants. But nowadays, it is not adequately available in the country due to deforestation problem in the years. In addition, its economic importance, current status, and medicinal roles are not well documented and understood. As for research studies, it is concluded that unless a collective effort is taken, the existence of this plant is under severe threat and needs to have some measures. This review article is aimed at addressing the abovelined topics in detail and to pinpoint and explain the importance and status of *Ximenia americana*.

## 1. Introduction

The history of agriculture showed that the transition from food collection to food production requires the domestication of plants and rearing of animals. Although there is no precise evidence about the origin of agriculture, Childe [1] proposed that a second Neolithic revolution coinciding with the Bronze Age occurred between 6000 and 3000 BC; the change from villages to urban communities is the source for the development of settled agriculture.

Fruit culture, starting from its onset which is during the earliest periods of man settlement mostly up to its modern production with so many sophisticated equipment and technologies, passes through selection, vegetative propagation, irrigation, pruning, training, pollination, harvesting, and storage [2, 3]. Through those processes, human ancestors developed their association with the fruit growing, and they thrive with some of them as food, medicinal, and animal feed roles [4].

The selection process favors some fruits to be domesticated as well as utilized and some others to be used by human beings as wild. Fewer than 20 plant species produce 90% of the food consumed by the global population [5]. Zemede and Mesfin [6] by referring different authors to various sources reported that about 5% of the total plant species of the world which serve for human beings are domesticated, used as food, and another supplement. The remaining plant species are wild, and most of them are utilized as wild.

Wild edible fruits are important in the economy, medicine, and forage [7–9]. Not only are they important in these aspects, besides they have a role in preserving cultural heritages and maintaining ecological balance [10]. In Ethiopia, it is common to consume wild fruit crops mostly in rural areas. These wild plants are important as a food supplement, means of survival during drought periods, as medicine, a source of fuelwood, as animal feed, and to protect soil erosion [11].

The Ethiopian flora is estimated to contain between 6500 and 7000 species of higher plants of which about 12% is endemic [12]. Reports indicated that about 8% of the nearly 7000 higher plants of the country are edible. Of these, 203 wild and semiwild plant species are documented [13]. But there are many other wild edible plants that are not documented. More recently, some ethnobotanical studies were undertaken in some parts of the country [12, 13]. However, the majority of these studies have dealt with medicinal species, and little emphasis has been paid to wild edible fruit plants.

In Ethiopia*, Ximenia americana* (local name Inkoy) is one of the wild fruit plants which are given little emphasis on its general characteristics, food value, medicinal value, and its climatic and management requirements. Even if local people know the use and management of *X. americana*, only a few research studies have been performed by few researchers such as Bahiru et al. [14] and Debela et al. [15]. Therefore, the importance of this review is to pinpoint and to explain importance and status of *Ximenia americana* in Ethiopia.

## 2. Botany of *Ximenia americana*

*Ximenia americana*, which belongs to family *Olacaceae* with common names English (hog plum, wild plum, and false sandalwood), Amharic (inkoy and kol), Tigrigna (mlehtta and mullo), is a semiscandent bush-forming shrub or small tree 2–7 m high [13]. Mostly, its trunk diameter is not greater than 10 cm; the color of the bark is dark brown to pale grey and smooth to scaly. Its branchlets are purple-red with a waxy bloom, and the tree is usually armed with straight slender spines. Sometimes, there is semiparasitic nature with haustoria on the roots. Its leaves are alternate, lanceolate to elliptic, 3–8 to 1.5–4 cm, variable thickness (semisucculent to thin), obtuse or emarginate, 3–7 pairs of veins, inconspicuous. Petioles are short, slender up to 6 mm long, and canaliculated, and it has grey-green, hairless, and leathery or thin flesh. When crushed, the young leaves have the smell of bitter almonds [16].

The flower colors are mainly white, yellow-green, or pink which are developed in branched inflorescences. These inflorescences are borne on pedunculate axillary racemes or umbels which are on pedicles 3–7 mm long. The fruits are globose to ellipsoidal drupes about 3 cm long, 2.5 cm thick, glabrous, greenish when young, become yellowish (or rarely orange-red) when ripe, contain a juicy pulp, and 1 seed. Its seed is woody, light yellow, up to 1.5 cm long, 1.2 cm thick with a fatty kernel, and a brittle shell. The species flowers and their fruit ripen throughout the year; flowering and fruiting periods do not seem to be governed by climatic regimes. Fruits are dispersed by animals [17].

It is diversified in different locations such as countrysides, savannah, forest lands, dry woodlands, and riverbanks. It is drought-resistant with an altitude requirement of 900–2,000 masl, mean annual temperature of 14–30°C, and mean annual rainfall of 300–1 250 mm. It prefers poor and dry soil, including clay, clay loam, loamy sand, sandy clay loam, and sand [17].

## 3. Importance of *Ximenia americana* in Different Parts of the World

Traditionally, *X. americana*' fruits and leaves have several uses in medication as folks for humans and animals [18]. The leaves and twigs of the plant are used for fever, cold, as a solution for toothaches, as a laxative and eye lotion, and poison cure [19–21]. The root part of the plant is also used as a treatment for skin burns, leprosy, headaches, hemorrhoids, guinea worm attack, sleeping thickness, puffiness, and some sexually transmitted diseases [22]. The fruit is eaten in quantities to treat constipation by eating heavy foods and vermifuges. The bark is used to treat skin ulcers, after some local processing systems of drying, powder making, and applied to skin ulcers. The fruits are eaten in large quantities and act as a vermifuge [23, 24]. The leaves are used for headaches. It is also indicated that *Ximenia americana* has a wide role in controlling many more different humans as well as animal diseases [20, 25].

*Ximenia americana* bark contains approximately 17% oils; heartwood and flowers contain essential oils [26]. The seeds and fruit pulp reportedly contain a substantial amount of hydrocyanic acid. The processed and extracted oil which is mainly edible, nondrying, is also suitable for soap preparation, as lubrication, traditionally as body anointing, and as a cure for chapped and dry feet pulp [27].

*Ximenia americana* oil is reported to have a higher amount of saturated and monounsaturated fatty acids (about 99%), which gave it stability to oxidation. Experiments performed on the oil indicated that it is very useful to treat dry skin which is prone to early senescence and enhances the activities of the sebaceous tissues. Moisturizing, softening, and revitalizing of skin is the main importance of unsaturated fatty acids extracted from the fruit of the plant [28].

Orally took *Ximenia americana* as fresh or processed form also works well for treating skin wounds, hemorrhoids, and helps with looseness of the bowels. In addition, it is a decent pain-relieving agent that lowers fevers; at the same time, it additionally works well to fight off viral infections such as measles [29]. Some of the compounds in this plant help with insulin-resistant diabetes, and for treating Alzheimer's disease, improving memory, and lowering cholesterol.

The fruit and the stems have powerful compounds which are anticancer, antifungal, antiallergic, antiparasitic, antiviral, and antibacterial, and it is also a very good antioxidant [30–32]. It is also indicated that it is effective in healing arthritis. A higher amount of healthy fatty acids are available in seeds, and the antibacterial capacity of the leaf extract is reported to be equal to penicillin and is effective for killing gonorrhea and cancer cells [32].

Shagal et al. [33] concluded that phytochemical screening of three different plant parts using ethanol and water as solvent showed that tannins, saponins, flavonoids, phenols, and volatile oils were present, and ethanol extracts of the plant parts were found to have antimicrobial activity on *S. aureus*. They also found that the water extract showed antimicrobial activity on *S. aureus* and *E. coli.*

Researchers around the world reported that the orange shaped fruit that tastes a little bit like almonds is pack full of vitamin C (vitamin C is a great antioxidant that helps to prevent cardiovascular disease, strokes, and cancer). *Ximenia* fruit reportedly contains an ample amount of vitamin E, minerals fiber, carbs, starches, and higher content protein as well. It also has nonessential proteins, and the stem, bark, and leaves of the tree also contain lots of natural steroids that may be used in the future for treating diseases such as cardiovascular disease and strokes [34–36].

This implies that the plant has a lot of benefits on the environment, food security, and medicinal value, input for industries, and if properly handled as well as invested on, it may be a source of income for developing countries such as Ethiopia.

## 4. Importance of *Ximenia americana* in Ethiopia

### 4.1. Local Medicine

In Ethiopia, the impact of traditional medicine on the health and wellbeing of the people is significantly high. Nearly 80% of the people are dependent on traditional medicine to treat different human and animal diseases. This could be due to the cost and nonavailability of modern drugs, lack of extended healthcare services, and higher acceptability of traditional medicines [37].

Ethnobotanical and ethnomedicinal studies in different parts of Ethiopia indicated that *Ximenia americana* plants have diverse use in the country. Culturally, the plant is utilized as a medicine for various animal and plant diseases. It is reported to give relief to stomach complaints, placenta expulsion [38], internal parasitism, worm infestations [39], treatment of hepatitis, and malaria (Table 1) [42].

It was also indicated that the local people of Dak Island use the bark of the plant as a medicine for rabies diseases. The process of utilization is by soaking the bark in the water and the water is taken orally [43]. The oils extracted from fruit kernel are also utilized as oil flesh wounds to prevent infections [44].

The screening of *Ximenia americana* extracts was tested for its antiviral activity for its inhibitory effects on the in vitro replication of HIV-1 and HIV-2. The results indicated that lower antiviral activity was recorded than the level necessary to produce cytotoxicity (CC50: 37.7 mg/mL). However, the result warrants further investigation because of the selectivity shown by the plant extract against HIV-1 and HIV-2 strains tested [45].

### 4.2. Food and Feed Value of *Ximenia americana* in Ethiopia

It is indicated that the plant is one of the main wild edible plants which is eaten during famine periods in most parts of the country. This indicated the availability of the plant while there is a drought for other plants [46]. It is also reported that *Ximenia americana* plant is one of the wild edible plants that could enhance food security in different parts of Ethiopia [47–49].

It is one of the most utilized fruits by livestock herders, children, women, and the poorest families. Its main utilization is as a food and some amount sold in the roads and local markets [17]. In different parts of Ethiopia, the leaf is highly important as a feed for cattle, goats, and sheep. The plant is available in all months of the year as it is not a deciduous plant. Hence, its leaf is available in all months of the year as a feed for animals [50]. In Tsemay and Benna ethnic groups of the country, the plant is reported to have a supplementary role as a household food security [51].

Shred of evidences also suggest that the plant is used as one of the many wild edible plants utilized as an emergency, supplementary, or seasonal food sources to avert food insecurity in rural households of Ethiopia [52].

The fruit of these plants is also reported to have an ample amount of protein, fiber, vitamins, lipids, and amino acids. Furthermore, it also contains the main essential minerals such as calcium, magnesium, potassium, sodium, iron, and manganese. The plant seeds also have low antinutrient content which could make them a good dietary supplement for both human and animal feeds formulation [53] (Table 2). Hence, consumption of this fruit in wild in the country could supplement and enhance the food consumption habit of the country.

### 4.3. Economic Value

The use of wild edible plants and their products in human life has grown from ancestral practices [53, 55, 56]. This could enhance household food security in wild plants consuming territories, especially in rural areas [54]. In Ethiopia, many wild edible plants have been consumed wild which enhances the food security status of households throughout the year. Especially, in the rural areas, there is a culture of consuming wild plants in pastoral, agropastoral, nomadic, and rural areas [56, 57].

*Ximenia americana* is one of the known fruits which is consumed in different parts of the country. It was indicated that the average production of a single *Ximenia americana* plant is near to 50.33 kg wet fruits, which can create an income amount of 2516.50 Ethiopian Birr which can be sufficient enough to buy main staple foods for a given household [58, 59]. Being one of the major plants in local medicine for humans and animals, it plays a significant role in reducing household expenditure in medicine costs [60].

The house making, fuelwood (charcoal and firewood), and in-house materials all over the country are still greatly dependent on forest and forest tree products. Different plants are selected for different purposes. *Ximenia americana* is one of the plants which is utilized as a source of firewood, fencing, and in preparing the housing products [17, 61].

The plant is economically important in other parts of the world. It is valuable in producing soup, oils, cosmetics, skin cream, medicinal tablets production, and as food [4, 24, 35]. But in Ethiopia, there is no company involved in the processing and producing *Ximenia americana* products. Therefore, except for consumption and local medicine use by local people, it is not yet under the industrial system.

## 5. Current Status of *Ximenia americana* in Ethiopia

In Ethiopia, this plant has been used by the rural people for a long time as a source of fresh fruit, as a medicine, and as a supplement for firewood. But there are few ethnobotanical studies and are limited on knowing its existence and thereby with few explanations about its importance [37].

*Ximenia americana* is also reported as one of the plants which face threats of extinction. The factors responsible as a cause are forest clearing, grazing, timber harvesting, charcoal making, drought and bark, and root harvesting. The most fearful thing about this plant is also the conservation status is very low as compared to other wild edible plants [46]. Previously, the plant was widely available in different parts of the country. But, nowadays due to its huge depletion and threat of extinction, it is not accessible even for local medicine purposes [37]. It could be associated with the climate change and other factors associated [15].

It is indicated that some national parks such as Awash National Park played a significant role in conserving wild edible fruits. *Ximenia americana* is one of the wild edible fruits which is available inside the park and scarce outside it [17]. This showed that even if the plant is not available in normal forest lands and faced extinction, its availability in national parks will be hope in future conservation practices.

**Figure 1:**
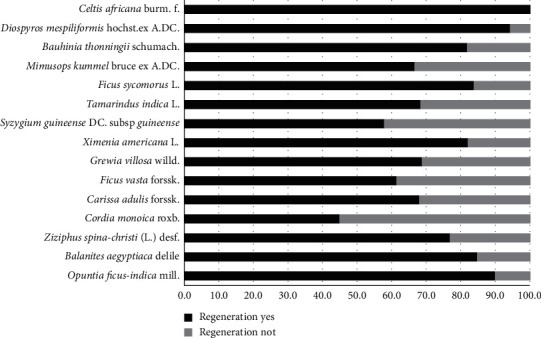
Regeneration status of *Ximenia americana* in low lands of Ethiopia. Source: Dejene et al. [46].

Dejene et al. [46] reported that the plant faces threats such as fire, clearing, grazing, timber harvesting, leaf harvesting, fruit harvesting, flower harvesting, root harvesting, bark harvesting, charcoal making, pest infestation, drought, and others. Whereas, the regenerative capacity of the plant is promising. The result indicated that the plant is among one having higher regenerative capacity among wild edible plants (Figure 1).

According to Debela et al. [15], unless the situation is reversed by a collective effort, there is a fear of extinction of this wild edible fruit in the near future particularly from the rate of deforestation, selective removal, and indiscriminate degradation of local habitat and expansion of grazing and farmlands. This report also sets the most important conservation options which can reverse the problem are sustaining multipurpose uses, rehabilitating, improving the ecosystem and enhancing its services, enhance resilience to climate change, promoting indigenous knowledge, diversify food sources, and biocultural heritage.

Yadev [44] also reported that wild edible fruits and other vegetative resources of the country are dwindling due to exploitation. To conserve these resources, systems such as medicinal gardens, proper handling practices, and scientific development are needed to be implemented.

## 6. Conclusion

*Ximenia americana* plants have economic, medicinal, and forage values; in addition, they preserve cultural heritages and maintain ecological balance by providing various ecosystem services. However, the plant faced threats of extinction due to forest clearing, grazing, timber harvesting, charcoal making, drought, and bark and root harvesting. And also, the conservation status is very low as compared to other wild edible fruit plants. Hence, the most important conservation options which can reverse the problem are sustaining multipurpose uses, rehabilitating, improving the ecosystem and enhancing its services, enhance resilience to climate change, promoting indigenous knowledge, and diversify food sources and biocultural heritage. In addition, to conserve this resource, systems such as medicinal gardens, proper handling practices, and scientific development are needed to be implemented.

## Figures and Tables

**Table 1:** Traditional medicinal uses and parts used of *Ximenia americana* in Ethiopia.

Use	Part of plant used	Ways of preparation	Authors
Lung abscess	Root and bark	Crushed, homogenized in water, and drunk	[40]
Muscle cramp	Root and bark	Crushed, homogenized in water, and drunk	[40]
Wounds	Root and bark	Crushed, homogenized in water, and drunk	[40]
Hepatitis	Seed and leaf	Crushed, homogenized in water, and drunk	[38]
Kidney problem	Fruit and leaf		[41]
Abdominal pain	Stem and bark	Crushed, homogenized in water, and drunk	[38]
Antivomit, leech infestation, and tonsillitis	Leaf		[17]

**Table 2:** Physicochemical properties of the oil extracted from *X. americana* pulp and seed.

Parameter	Pulp	Seed
Acid value	0.29 ± 0.15	0.56 ± 0.15
Saponification value (mgKOH/g oil)	178.12 ± 0.02	179.94 ± 1.69
Peroxide value (mEq O_2_/kg)	27.80 ± 0.11	30.06 ± 0.12
Iodine value (g I2/100 g)	43.17 ± 0.25	40.61 ± 0.10
FFA (%)	0.15 ± 0.10	0.29 ± 0.15
Refractive index	1.4330 ± 00	1.4130 ± 00
Specific gravity	0.8980 ± 00	0.9493 ± 00
Viscosity (25°C) (g/cm^3^s^−1^)	46.99 ± 0.15	48.00 ± 0.01
Color	Light brown	Light brown

Source: Tanko et al., [54].

## Data Availability

No primary data were used to support this study.

## References

[1] Childe V. G. (1958). *The Down of European Civilization*.

[2] Neilsen D. (2005). Fundamentals of temperate zone tree fruit production. *Tree Physiology*.

[3] Harford R. (2011). *Eat weeds. Wild Food Guide to the Edible Plants of Britain*.

[4] Benoit V. F., Santillana H. M., Kone B. D., Mallie M., Derouin F. (2000). Anti-toxoplasma activity of vegetable extract used in West Africa traditional medicine. *Parasite*.

[5] Maheshwari J. K. (1988). Ethnobotanical research and documentation. *Acta Universitatis. Upsalienses Symbole Botanicae Upsalienses*.

[6] Zemede A., Mesfin T. (2001). Prospects for sustainable use and development of wild food plants in Ethiopia. *Economic Botany*.

[7] Sardeshpande M., Shackleton C. (2019). Wild edible fruits: a systematic review of an under-researched multifunctional NTFP (non-timber forest product). *Forests*.

[8] Addis G., Asfaw Z., Woldu Z. (2013). The role of wild and semi-wild edible plants in household food sovereignty in Hamer and Konso communities, South Ethiopia. *Ethnobotany Research and Applications*.

[9] Deshmukh B. S., Waghmode A. (2011). Role of wild edible fruits as a food resource: traditional knowledge. *Explore Research Article [Deshmukh Waghmode International Journal of Pharmacy Life Science*.

[10] Fantahun F. M. T., Hager H. (2009). Exploiting locally available resources for food and nutritional security enhancement: wild fruits diversity, potential and state of exploitation in the Amhara region of Ethiopia. *Food Security*.

[11] Gebre Egziabher T. B., Engles J. M. M., Hawkes J. G., Worede M. (1991). Diversity of the Ethiopian flora. *Plant Genetic Resources of Ethiopia*.

[12] Tamene B. (2000). A floristic analysis and ethnobotanical study of the semi-wet land of Cheffa area, South Welo, Ethiopia.

[13] Gemedo D., Brigitte L., Maass J. I. (2005). Plant biodiversity andethnobotany of borana pastoralists in southern oromia, Ethiopia. *Economic Botany*.

[14] Bahru T., Zemede A., Sebsbe D. (2013). Wild edible plants: sustainable use and management by indigenous communities in and the buffer area of Awash national park, Ethiopia. *Ethiopian Journal of Science*.

[15] Debela H., Jesse T. N., Zemede A., Nyangito M. M. (2012). Uses and management of *ximenia americana, olacaceae* in Semi-arid east shewa, Ethiopia. *Pakistan Journal of Botany*.

[16] Maundu P. M., Ngugi G. W., Kabuye C. H. S. (1999). *Traditional Food Plants of Kenya, Kenya Resource Centre for Indigenous Knowledge*.

[17] Sacande M., Vautier H. (2006). *Ximenia Americana. Seed Leaf Let No. 112, April 2006*.

[18] Mwangi J. W., Malii P., Gathu L., Tanaka T., Nonaka G. (1994). Polyphenols of *Ximenia americana*. *Fitoterapia*.

[19] De Menezes I. R. A., Da Costa R. H. S., Augusti Boligon A. (2019). *Ximenia americana* L. enhances the antibiotic activity and inhibit the development of kinetoplastid parasites. *Comparative Immunology, Microbiology and Infectious Diseases*.

[20] Le N. H. T., Malterud K. E., Diallo D., Paulsen B. S., Nergård C. S., Wangensteen H. (2012). Bioactive polyphenols in *Ximenia americana* and the traditional use among Malian healers. *Journal of Ethnopharmacology*.

[21] Feiberger C. E., Vanderjagt D. J., Pastuszyn A. (1998). Nutrient content of the edible leaves of seven wild plants from Niger, Plant foods human nutr. *Dardrecht*.

[22] Teo S. P. (1997). Root hemi parasitism in Malayan olecaceae. *Gardens’ Bulletin Singapore*.

[23] Kuroki G. W., Conn E. E. (1989). Mandelonitrile lyase from Ximenia americana L.: stereospecificity and lack of flavin prosthetic group. *Proceedings of the National Academy of Sciences*.

[24] Niemi L., Wennström A., Ericson L. (2005). Insect feeding preferences and plant phenolic glucosides in the system Gonioctena linnaeana—salix triandra. *Entomologia Experimentalis et Applicata*.

[25] Omer M. E. A., Ali M. A. Z. (1998). Sudanese plants used in folkloric medicine screening for antimicrobial activity. *Fitoterapia*.

[26] Fatope M. O., Adam O. A. (2005). C18 acetylene fatty acids of *Ximenia americana* with potential pesticidal activity. *Journal Agriculture Food Chemistry*.

[27] Sallamander C. (2010). Ximeniaoil. esoteric oils CC, Sallamander concepts (Pty) Ltd 1998–2010. http://www.essentialoils.co.za.htm.

[28] Sarmento J. D. A., Dantas de Morais P. L., Israël de Souza F., Alcântara de Miranda M. R. (2015). Physical-chemical characteristics and antioxidant potential of seed and pulp of Ximenia americana L. from the semiarid region of Brazil. *African Journal Biotechnology*.

[29] Koné W. M., Atindehou K. K., Terreaux C., Hostettmann K., Traoré D., Dosso M. (2004). Traditional medicine in North Côte-d’Ivoire: screening of 50 medicinal plants for antibacterial activity. *Journal of Ethnopharmacology*.

[30] Maikai V. A., Maikai B. V., Kobo P. I. (2009). Antimicrobial properties of stem bark extracts of *Ximenia americana*. *Journal of Agriculture Science*.

[31] Omer M. E. F. A., Elnima E. I. (2003). Antimicrobial activity of Ximenia americana. *Fitoterapia*.

[32] Anonymous (2010). (world agroforestry centre). Ximenia Americana. tree species reference and selection guide, agroforestry data base. http://www.worldagroforestry.org.

[33] Shagal D. M. H., Kubmarawa J., Barminas J. T. (2013). Evaluation of antimicrobial property of *Ximenia American*. *Journal of Biotechnology and Pharmaceutical Research*.

[34] Hou X.-L., Takahashi K., Tanaka K. (2008). Curcuma drugs and curcumin regulate the expression and function of P-gp in Caco-2 cells in completely opposite ways. *International Journal of Pharmaceutics*.

[35] Oladipo G. O., Eromosele I. C., Folarin O. M. (2013). Formation and characterization of paint based on alkyd resin derivative of ximenia americana (wild olive) seed oil. *Environment and Natural Resources Research*.

[36] Lucilania M. B. A., Wallace F. D. S. L., Patrícia L. D. M., Dárcio J. S. A., Elesbão R. (2016). “Bioactive compounds and antioxidant potential fruit of ximenia Americana L. *Food Chemistry*.

[37] Desissa D., Binggeli P. (2020). Uses and conservation status of medicinal pants used by the Shinasha people. http://www.mikepalmer.co.uk/woodyplantecology/ethiopia/shinasha.htm.

[38] Abate G., Demissew S. (1989). Etse Debdabe: Ethiopian Traditional Medicine. *Ethiopian traditional medicine*.

[39] Getahun A. (1976). *Some Common Medicinal and Poisonous Plants Used in Ethiopian Folk Medicine*.

[40] Wondimu T., Asfaw Z., Kelbessa E. (2006). Ethnobotanical study of food plants around ’ dheeraa ’ town , arsi , Ethiopia. *African Journals*.

[41] Jansen P. C. M. (1981). Spices, condiments and medicinal plants in Ethiopia, their taxonomy and agricultural signifi cance.

[42] Feyssa D. H., Njoka J. T., Asfaw Z., Nyangito M. M. (2012). Uses and management of *Ximenia americana*, olacaceae in semi-arid east Shewa, Ethiopia. *Pakistan Journal of Botany*.

[43] Teklehaymanot T. (2009). Ethnobotanical study of knowledge and medicinal plants use by the people in dek island in Ethiopia. *Journal of Ethnopharmacology*.

[44] Yadav Hiranmai R. (2013). Medicinal plants in folk medicine system of Ethiopia. *Journal of Poisonous and Medicinal Plants Research*.

[45] Asres K., Bucar F., Kartnig T., Witvrouw M., Pannecouque C., De Clercq E. (2001). Antiviral activity against human immunodeficiency virus type 1 (HIV-1) and type 2 (HIV-2) of ethnobotanically selected ethiopian medicinal plants. *Phytotherapy Research*.

[46] Dejene T., Mohamed S. A., Dolores A., Pablo M. P. (2020). Ethnobotanical survey of wild edible fruit tree species in lowland areas of Ethiopia. *Forests*.

[47] Getahun A. (1974). The role of wild plants in the native diet in Ethiopia. *Agro-Ecosystems*.

[48] Gelmesa D. (2010). *Shifting to Alternative Food Source: Potential to Overcome Ethiopians’ Malnutrition and Poverty Problems. Innovation and Sustainable Development in Agriculture and Food (ISDA). Montpellier*.

[49] Hunde D., Njoka J. T., Nyangito M. M., Zemede A. (2010). Neutraceutical wild plants of semiarid east Shewa, Ethiopia: contributions to food and healthcare security of the semiarid people. *Research of Journal Forest*.

[50] Aregawi T., Melaku S., Nigatu L. (2020). Management and utilization of browse species as livestock feed in semi-arid district of north Ethiopia. *Livestock Research for Rural Development*.

[51] Assefa A., Abebe T. (2010). Wild edible trees and shrubs in the semi-arid lowlands of southern Ethiopia. *Journal of Science & Development*.

[52] Lulekal E., Asfaw Z., Kelbessa E., Van Damme P. (2011). Wild edible plants in Ethiopia: a review on their potential to combat food insecurity. *Afrika Focus*.

[53] Suwardi A. B., Navia Z. I., Harmawan T., Syamsuardi, Mukhtar E. (2020). Wild edible fruits generate substantial income for local people of the gunung leuser national park, aceh tamiang region. *Ethnobot Research Application*.

[54] Tanko E., Ajai A. I., Lafiya-Araga R. A., Dauda B. E. N., Mathew J. T., Omozokpia J. (2017). Physico-chemical, fatty acid profile and amino acid composition of the fruit pulp and seeds of ximenia americana L. (Tallow plum) obtained in Niger state. *Niger International Journal of Food Chemistry*.

[55] Ong H. G., Kim Y.-D. (2017). The role of wild edible plants in household food security among transitioning hunter-gatherers: evidence from the Philippines. *Food Security*.

[56] Seble W. Y., Asfaw Z., Kelbessa E. (2015). Ethnobotanical study of medicinal plants used by local communities of minjar-shenkora district, north shewa zone of amhara region, Ethiopia. *Journal of Medicinal Plants Research*.

[57] Zone A., Wondimu T., Asfaw Z., Kelbessa E. (2007). Ethnobotanical study of medicinal plants around. *National Library of Medicine*.

[58] Ounyambila L., Anne M. L., Benjamin L., Amadé O. (2018). Influence of climate on fruit production of the yellow plum, Ximenia americana, in Burkina Faso, West Africa. *Journal of Horticulture and Forestry*.

[59] Hariramamurthi G., Venkatasubramaniam P., Unnikrishnan P. M., Shankar D., Burford (2007). Home herbal gardens-A novel health security strategy based on Peopleʼs knowledge and resources. *Gerard Bodeker and Gemma*.

[60] Alemayehu G., Asfaw Z., Kelbessa E. (2015). Plant diversity and ethnobotany in berehet district , north shewa zone of amhara region (Ethiopia) with emphasis on wild edible plants. *Journal of Medicinal Plants Studies*.

[61] Zenebe G., Zerihun M., Solomon Z. (2012). An ethnobotanical study of medicinal plants in asgede tsimbila district, northwestern tigray, northern Ethiopia. *Ethnobotany Research and Applications*.

